# Effect of Microfluidization Technique on the Physicochemical Characteristics of Cannabidiol Nanoemulsions

**DOI:** 10.3390/nano16080459

**Published:** 2026-04-14

**Authors:** Andrés Fernando Sánchez Martínez, Luis Eduardo Diaz Barrera, Natalia Elizabeth Conde Martínez, Rosa Helena Bustos Cruz, Martha Ximena León Delgado, María Ximena Quintanilla Carvajal

**Affiliations:** 1 Grupo de Investigación de Procesos Agroindustriales, Facultad de Ingeniería, Universidad de La Sabana, Km 7 Vía Autopista Norte, Bogotá 250001, Colombia; andressama@unisabana.edu.co; 2 Grupo de Investigación en Bioprospección, Facultad de Ingeniería, Universidad de La Sabana, Km 7 Vía Autopista Norte, Bogotá 250001, Colombia; luisdb@unisabana.edu.co (L.E.D.B.); natalia.conde@unisabana.edu.co (N.E.C.M.); 3 Evidence-Based Therapeutics Group, Department of Clinical Pharmacology, Faculty of Medicine, Universidad de La Sabana and Clínica Universidad de La Sabana, Km 7 Vía Autopista Norte, Bogotá 250001, Colombia; rosa.bustos@unisabana.edu.co; 4 Grupo Dolor y Cuidados Paliativos, Facultad de Medicina, Universidad de La Sabana, Km 7 Vía Autopista Norte, Bogotá 250001, Colombia; martha.leon@unisabana.edu.co; 5 Engineering Department, Universidad de La Sabana, Km 7 Vía Autopista Norte, Bogotá 250001, Colombia; 6 Unisabana Center for Translational Science, School of Medicine, Universidad de La Sabana, Km 7 Vía Autopista Norte, Bogotá 250001, Colombia

**Keywords:** microfluidization, cannabidiol, nanoemulsions, HaCaT, nanoencapsulation, cytotoxicity, nanoparticles

## Abstract

This study examines the effect of microfluidization on the physicochemical properties, stability, release behavior, and cytocompatibility of cannabidiol (CBD) nanoemulsions intended for topical application. CBD is a non-psychoactive cannabinoid characterized by anti-inflammatory and analgesic activity; however, its therapeutic use is limited by low solubility and poor bioavailability. To address these limitations, nanoemulsions were formulated using avocado oil and Tween 80 and optimized through a Box–Behnken experimental design evaluating microfluidization pressure (5000–20,000 PSI), CBD concentration (0–2%), and oil content (8–10%). Nanoemulsions were characterized over a 60-day period in terms of droplet size, dispersity index (D), and zeta potential. An increase in processing pressure led to a reduction in both droplet size and dispersity, with optimal conditions identified between 11,000 and 15,000 PSI. Higher oil and CBD concentrations were associated with an increase in the magnitude of the zeta potential, contributing to electrostatic stabilization of the system. Encapsulation efficiency reached approximately 81.4%. Cell viability assays in HaCaT keratinocytes indicated no significant cytotoxic effects. The optimized formulation exhibited a sigmoidal CBD release profile best described by Weibull and Gompertz models (R^2^ ≈ 0.99), suggesting combined diffusion and interfacial mechanisms that support efficient topical delivery.

## 1. Introduction

Musculoskeletal disorders affect individuals of all ages, and in 2019, it was reported that approximately 1.71 billion people worldwide suffer from musculoskeletal system disorders. The diagnosis and type of musculoskeletal disorders depend on age and environmental factors; however, these conditions affect a broad population across different age groups [1]. One of the most noticeable and perceptible external symptoms of musculoskeletal discomfort is inflammation. This is a process through which the body’s immune system recognizes and eliminates harmful or foreign stimuli, thereby initiating the healing process [2].

Currently, various products are available for muscle recovery and localized relief, with nonsteroidal anti-inflammatory drugs (NSAIDs) being among the most commonly prescribed treatments. However, these agents have been associated with hypersensitivity reactions in approximately 5.7% of patients, attributed to immune-mediated responses, with durations ranging from days to several years. In response, alternative formulations based on compounds of natural origin have been developed using different extraction approaches as potential substitutes for NSAIDs [3]. However, these alternative treatments are frequently marketed over the counter (OTC) and through online platforms, where variability in manufacturing practices and quality control may result in the presence of impurities or contaminants with potential adverse effects. Among the compounds used as alternatives to NSAIDs, eucalyptol is commonly incorporated due to its topical applicability and reported antibacterial activity, along with a relatively low incidence of contraindications [4]. However, when ingested, this compound can pose substantial health risks [5].

**Figure 1:**
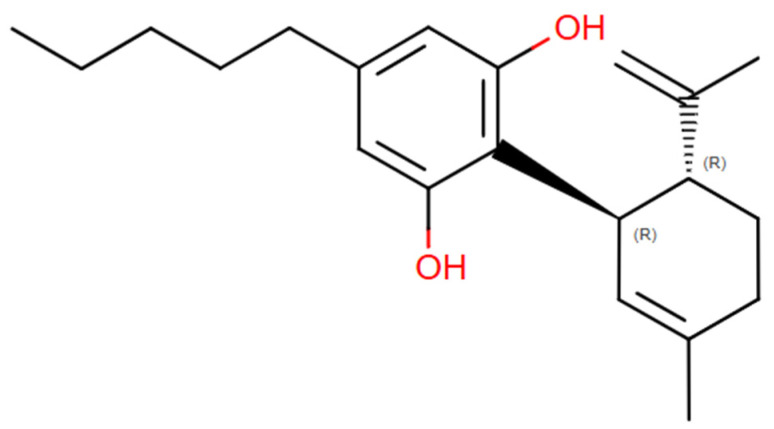
Molecular structure of CBD drawn using the ACDLabs program.

As an alternative, the cannabis plant contains components of considerable interest for the development of functional products. These components, known as cannabinoids, include compounds with both analgesic and anti-inflammatory properties, as well as others with psychoactive effects [6]. The cannabinoid cannabidiol (CBD) in Figure 1 is found in greater proportion than delta-9-tetrahydrocannabinol (Δ9-THC). Despite their molecular similarities, their effects on endocannabinoid receptors differ significantly [7]. THC is a partial agonist of CB1 and CB2 receptors, leading to psychoactive effects in the brain and anti-inflammatory actions in the immune system. In contrast, CBD modulates receptor activity without direct binding, affects serotonin and adenosine receptors, and inhibits fatty acid amide hydrolase 1 (FAAH), thereby increasing anandamide levels [8]. This contributes to its anti-inflammatory and neuroprotective effects without causing psychological activity [9]. Currently, several products incorporating cannabidiol oil are marketed for pain relief [10], either topically or orally. However, when cannabidiol is ingested, the molecule undergoes pharmacokinetic changes, increasing the likelihood of Δ9-THC formation within the body [11].

Orally consumed CBD-based products have a 10% probability of inducing adverse effects [12]. Potential side effects of CBD ingestion include somnolence, diarrhea, reduced appetite, fatigue, vomiting, fever, lethargy, seizures, upper respiratory tract infections, and abnormal liver function test results [13]. Additionally, the integration of these products into metabolic pathways can hinder effective pharmacological interactions with other medications, potentially leading to severe adverse effects and treatment failures [12].

For this reason, the use of topical products is recommended; however, not all active compounds are able to penetrate the skin. Effective skin permeation depends on molecular size and physicochemical properties, typically favoring molecules in the nanometer scale rather than macromolecular ranges. Conventional products often fail to meet this molecular size requirement, thus limiting their anti-inflammatory potential [14]. Therefore, it is essential to design, characterize, and control the particle size of topical formulations made from cannabidiol nanoemulsions [15].

Particle size requirements vary depending on the intended application and target tissue, adhering to the principle that smaller particle sizes increase the likelihood of permeating the dermal membranes [16]. The optimal particle diameter for effective penetration of dermal layers falls within the nanometer scale [17]. To introduce controlled-sized CBD molecules into the body via a topical rather than oral route, an encapsulation process is necessary [18]. Several methods are available for nanoencapsulating active compounds like CBD, including solvent evaporation, spray drying, ultrasonication, and microfluidization [19]. While ultrasonication effectively reduces particle size, it is not a scalable process. In contrast, microfluidization not only reduces particle size but also lowers energy costs, making it highly efficient at an industrial scale [20]. Microfluidization is a high-energy process in which the oily and aqueous phases are combined with the active compound under high-pressure air, forming more efficient nano-level delivery systems [21]. Microfluidization offers several advantages in the development of nano delivery systems, specifically enabling the creation of stable nanoemulsions with particle sizes around 160 nm [22].

Therefore, this study aimed to investigate the effect of the microfluidization technique on the design and control of the physicochemical characteristics of cannabidiol nanoemulsions and its effect on HaCaT cells.

## 2. Materials and Methods

### 2.1. Materials

Cannabidiol (CBD) crystals were provided by CLEVER LEAVES (Florida, USA and Tocancipá, Cundinamarca, Colombia). The CBD crystals were shipped from the U.S. headquarters and subsequently received at the facilities located in Tocancipá, Cundinamarca. Upon arrival, the samples were transported to Universidad de La Sabana, where they were stored under controlled conditions in accordance with the guidelines established in Resolution 227 of 2022 by the Ministries of Justice, Agriculture and Rural Development, and Health and Social Protection.

Tween 80 was selected as the surfactant based on its emulsifying capacity and food-grade classification and was supplied by SUQUIN S.A.S. (Bucaramanga, Santander, Colombia). Avocado oil was obtained from GRUPO NUTRESA S.A. (Medellin, Colombia). According to its technical datasheet, the main fatty acid composition of the avocado oil consisted of Palmitic Acid (28.21%), Palmitoleic Acid (5.69%), Stearic Acid (0.70%), Oleic Acid (50.95%), Linoleic Acid (13.87%), and Linolenic Acid (0.58%). For the in vitro assays, HaCaT cells were used, cultured in Dulbecco’s Modified Eagle Medium (DMEM, Gibco, Thermo Fisher Scientific, CA, USA) supplemented with 10% Fetal Bovine Serum (FBS, Gibco, Thermo Fisher Scientific, CA, USA) and 1% penicillin-streptomycin (Gibco, Thermo Fisher Scientific, CA, USA). The cytotoxicity evaluation was performed using the MTT reagent (3-(4,5-Dimethylthiazol-2-yl)-2,5-Diphenyltetrazolium Bromide, Sigma-Aldrich, MA, USA), and for solubilization of formazan crystals, Dimethyl Sulfoxide (DMSO, Sigma-Aldrich, USA) was used. For chromatographic analyses, HPLC-grade Methanol (Sharlau, Barcelona, Spain) and HPLC-grade Water (Sharlau, Barcelona, Spain) were employed as mobile phase solvents.

### 2.2. Experimental Design

A Box–Behnken experimental design was employed to evaluate the effect of three factors on the nanoemulsion formulation: microfluidization pressure (5000–20,000 PSI) (A), CBD concentration (0–2% *w*/*w*) (B), and avocado oil concentration (8–10% *w*/*w*) (C). The response variables measured in this study included droplet size (nm), zeta potential (ζ), and dispersity (D). The objective was to determine the optimal composition and processing pressure that would ensure the desired stability and physicochemical characteristics of nanoemulsions. The experimental conditions were maintained over a period of 60 days, with measurements of dispersity, droplet size, and zeta potential being taken at regular intervals.

### 2.3. Development of Nanoemulsion

The preparation of nanoemulsions was initiated through a pre-emulsification stage involving the formation of coarse macroemulsions. The aqueous phase was formulated by dissolving polysorbate 80 (Tween 80) in distilled water at a concentration of 3% *w*/*w*, as previously optimized in preliminary assays (Supplementary Material). This phase was thermally conditioned to 65 °C using a temperature-controlled heating plate (Heidolph, Schwabach, Germany), ensuring adequate reduction in interfacial tension and viscosity. Simultaneously, high-shear homogenization was applied using an Ultra-Turrax T-18 disperser (IKA, Bogotá, Colombia) operating at 11,000 rpm to promote initial droplet disruption.

In parallel, the oil phase was prepared by solubilizing cannabidiol (CBD) crystals in avocado oil under controlled thermal conditions (~70 °C) to ensure complete dissolution and molecular dispersion. The oil phase composition was systematically varied, with avocado oil concentrations ranging from 8% to 10% *w*/*w* and CBD content between 0% and 2% *w*/*w*. Upon achieving a homogeneous lipid phase, it was incorporated into the aqueous phase via controlled dropwise addition at a constant flow rate over a period of 2 min, facilitating progressive interfacial adsorption of the surfactant and minimizing premature coalescence.

Subsequently, the resulting coarse emulsion was subjected to continued high-shear homogenization for 10 min at 65 °C to enhance droplet size reduction and improve dispersion uniformity. This step ensured the formation of a kinetically stable macroemulsion prior to high-pressure processing. An aliquot was collected at this stage to serve as a baseline sample prior to microfluidization, enabling comparative assessment of droplet size distribution and physicochemical properties following downstream processing [23].

To optimize the microfluidization process, the effect of cycle number on emulsion stability was evaluated. Preliminary experiments indicated that over-processing led to excessive destabilization of the emulsion. Based on these results, a cycle number of 2 was selected, as it produced emulsions with acceptable stability levels without requiring excessive energy input. The microfluidization pressure range was set between 5000 and 20,000 PSI. Following this, the macroemulsion was processed in an LM10 Microfluidizer (Microfluidics, Cambridge, UK), with two samples of approximately 20 mL each being taken. After processing, one sample was refrigerated, and the other was kept at room temperature for subsequent stability analysis.

### 2.4. Stability Study

The stability of the nanoemulsions was evaluated by monitoring four key parameters: zeta potential (mV), droplet size (nm), dispersity index (D), and phase separation. Characterization was conducted at five time points (0, 8, 15, 30, and 60 days). Samples were stored under two temperature conditions (8 °C and 20 °C) to assess the effect of temperature on system stability. For analysis, the samples were diluted with deionized water at a ratio of 1:1000 before measurements were taken using a Zetasizer NanoZS (Malvern Instruments, Cambridge, UK). The Zetasizer was equipped with a laser diffractometer, and measurements were performed at 25 °C. Each experimental run was performed in triplicate to ensure reproducibility and reliability of the results [24].

To further assess phase separation and other physical changes, samples were transferred to Falcon tubes for visual inspection. During the stability study, photographs of the samples were taken at each time point to document any observable changes in phase behavior [25].

### 2.5. Determination of Encapsulation Efficiency

Encapsulation efficiency (EE) was determined by adapting the methodologies of [26,27], with specific modifications to improve phase separation and analytical robustness. Briefly, 15 mL aliquots of the nanoemulsion were subjected to refrigerated centrifugation at 10,000 rpm for 30 min at 4 °C (Thermo Fisher Scientific Inc., CA, USA) to induce controlled destabilization and partial demulsification. This process enabled the formation of a translucent supernatant lipid phase, which was carefully recovered using positive displacement micropipettes to minimize interfacial disruption and avoid cross-contamination [27].

The recovered oil phase, containing the lipophilic CBD, was subsequently diluted in the chromatographic mobile phase to ensure complete solubilization and compatibility with the analytical system [15]. Quantitative analysis was performed by high-performance liquid chromatography (HPLC) using a SOLAS 100 Å C18 reversed-phase column (150 mm × 4.6 mm, 5 µm) [28]. The mobile phase consisted of methanol and water (45:55, *v*/*v*), delivered under isocratic conditions at a flow rate of 0.7 mL/min [29]. The column temperature was maintained at 40 °C, and detection was carried out at 214 nm. A 5 µL injection volume was used for both standards and samples, with a total runtime of 23 min to ensure adequate resolution and system suitability [30].

A cannabidiol (CBD) standard stock solution (1 mg/mL) was prepared by dissolving 1 mg of pure CBD in methanol and adjusting to a constant final volume. From this stock solution, a series of dilutions were prepared to construct the external calibration curve, yielding standard solutions with final concentrations of 0.008, 0.010, 0.015, 0.020, 0.030, and 0.040 mg/mL [31]. Quantification was performed using linear regression of peak area as a function of concentration. The encapsulation efficiency was calculated according to the following expression:

%Efficiency = Mquantified/Minitial × 100(1)

where Mquantified is the amount of CBD found in the oil phase and Minitial is the total CBD added during formulation.

### 2.6. Thermal Behavior of Optimal Nanoemulsion

The thermal behavior of the optimized CBD nanoemulsion was evaluated using Differential Scanning Calorimetry (DSC). A Mettler Toledo DSC (OH, USA) was employed for the analysis. Approximately 10 mg of the sample was placed in the DSC cell, and the temperature was gradually increased from 20 °C to 180 °C at a heating rate of 10 °C/min under a nitrogen atmosphere. The resulting thermograms provided insights into the thermal stability and phase transitions of the emulsion [32].

### 2.7. Release Kinetics

A Franz cell with an 11 μm membrane was adapted. The nanoemulsion was deposited on the upper section of the membrane to constantly contact by gravity [33]. Additionally, the entire assembly was carried out in an isothermal process at approximately 37 °C. The receiving medium was a mixture of propylene glycol and 7:3 ethanol maintained at a temperature and at a stirring speed of 360 RPM. Samples were taken every 15 min and measured in a UV-Visible spectrophotometer at 209 nm.

### 2.8. Cell Viability

The cytotoxicity and cell viability of the CBD nanoemulsion were assessed using the HaCaT cell line, which was obtained from Cytion (Mannheim, Germany). The cells were seeded at a density of 3 × 10^4^ cells per well in a 96-well plate. After 24 h, the medium was replaced with fresh medium, and the treatments consisting of the optimal CBD nanoemulsion and control systems were added to the wells at a final product concentration of 10%. After 24 h of exposure, the medium was removed, and 50 μL of MTT solution was added to each well. The cells were incubated for an additional 24 h at 37 °C with 5% CO2. The supernatants were then discarded, and the formazan crystals were dissolved in 100 μL of 10% dimethyl sulfoxide (DMSO). Absorbance was measured at 595 nm using a microplate reader [34].

### 2.9. Statistical Data Analysis

Statistical analysis was performed using Design Expert software Version 10.0.7 (Stat-Ease Inc., MN, USA). An Analysis of Variance (ANOVA) was conducted to evaluate the effects of CBD concentration, avocado oil concentration, and microfluidization pressure on the stability and physicochemical properties of nanoemulsions. The significance of the factors was assessed at a 95% confidence level. The results were analyzed using quadratic and linear models, and response surface methodology was used to optimize the experimental conditions.

## 3. Results and Discussion

### 3.1. Stability Study

Nanoemulsions obtained with 1 and 2 cycles of microfluidization were evaluated to analyze the effect of the number of cycles on the product’s stability, regardless of the factors evaluated in the experimental design. The stability study was conducted under two storage conditions: room temperature (20 °C) and refrigeration (4 °C), to determine whether storage temperature significantly affects the nanoemulsion stability [35]. Various destabilization phenomena may occur in this type of product, making it necessary to assess the potential for creaming—where a visible layer forms on the surface of the product—or complete phase separation [36].

Sampling was performed on days 0, 8, 15, 30, and 60 to monitor changes in droplet size, dispersity (D), and zeta potential. Table A1 presents the corresponding results for each nanoemulsion under both 1 and 2 cycles of microfluidization at 20 °C.

As shown in Table A1, nanoemulsions 4, 10, and 17 exhibited apparent stability in terms of zeta potential. However, it is important to note that the system exhibits a high energy state following microfluidization, which may result in zeta potential values outside the typical colloidal stability range (±30 mV).

In terms of dispersity, it is evident that emulsions processed at lower pressures exhibited significantly higher values. This suggests that the applied energy input was insufficient to effectively disrupt the droplets, leading to a broader size distribution [37]. Pressure plays a critical role in ensuring the homogeneity of the droplet size distribution, as lower pressures result in less energy transfer, leading to poorer droplet break-up and thus broader distributions [38].

Higher pressures resulted in smaller droplet sizes due to enhanced droplet disruption [39]. Table A1 also reveals that some nanoemulsions experienced further droplet size reduction after recirculation within the microfluidizer. This suggests that two cycles of microfluidization offer more effective processing, breaking droplets more uniformly and thereby reducing their size. It is well established that recirculation enhances emulsification and contributes to more significant droplet size reduction [40].

The dispersity values were also strongly influenced by the number of microfluidization cycles. In many cases, dispersity decreased after the second cycle. Recirculation leads to additional droplet breakup, producing a narrower droplet size distribution and, thus, lower dispersity values closer to 0.2—a threshold generally associated with good colloidal stability and low risk of phase separation [41]. Although dispersity values in Table A1 are generally low, nanoemulsions subjected to two cycles often showed even lower dispersity values, indicating greater homogenization. This effect was especially evident in nanoemulsion 7, which had a dispersity of 0.123. These results support the notion that increasing the number of cycles enhances system uniformity and reduces droplet size variability [42].

Zeta potential values were consistently negative due to the interfacial charge of the droplets forming the nanoemulsion [43]. At lower pressures, the lower degree of droplet disruption may result in higher surface charge accumulation, increasing electrostatic repulsion and improving system stability [44]. As shown in Table A1, the zeta potential values confirm this negative surface charge and indicate the presence of repulsive interactions between droplets [45]. Interestingly, emulsions subjected to only one cycle showed more negative zeta potentials compared to those processed through two cycles. This could be explained by better droplet homogenization during the second cycle, which reduces surface charge variation and thus the absolute value of zeta potential [46].

**Figure 2:**
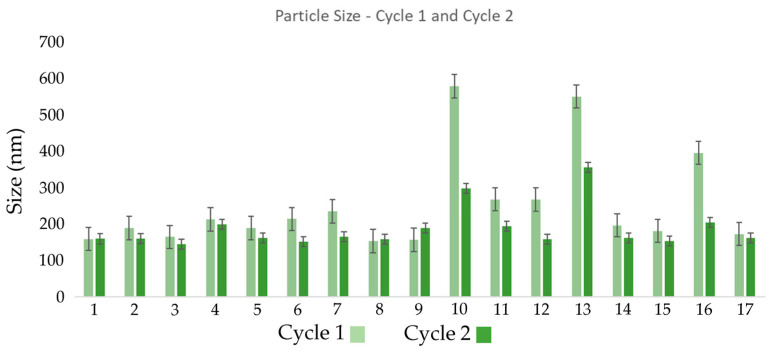
Graphical Analysis of Droplet Size Distribution at 20 °C for the evaluated formulations in cycle 1 and cycle 2. Error bars represent standard deviation.

Figure 2 clearly illustrates that recirculation within the microfluidizer influenced droplet size distribution in most runs. In certain cases, an increase in droplet size was observed after the second cycle, which may be attributed to the presence of higher solid content in the system. Additionally, particle size remained relatively constant between cycles for many formulations, suggesting that the first microfluidization cycle was already effective in producing adequately small droplets [47]. However, in some emulsions, improvements were observed after the second cycle, indicating that additional recirculation could enhance homogenization and further reduce particle size in specific cases [48].

In some formulations, the droplet size increased after the second cycle. This may be due to recoalescence, a phenomenon where additional mechanical energy favors fusion of smaller droplets into larger ones [49]. This may also be related to an insufficient concentration of Tween 80, the non-ionic surfactant responsible for stabilizing oil droplets in the aqueous phase. Tween 80 was selected for its ability to reduce interfacial tension and prevent coalescence. However, if its concentration is not adequate, particularly after a second cycle, the newly generated droplets may not be sufficiently coated, increasing the likelihood of fusion [50].

**Figure 3:**
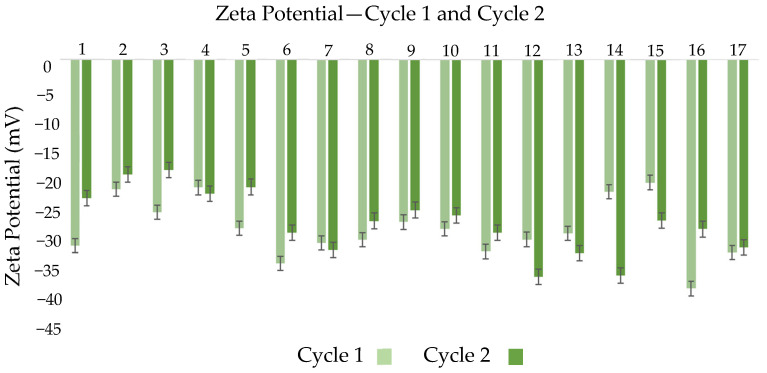
Graphical Analysis of Zeta Potential Distribution at 20 °C for the evaluated formulations in cycle 1 and cycle 2. Error bars represent standard deviation.

The zeta potential behavior presented in Figure 3 supports this observation. For most formulations, an additional cycle under high pressure altered zeta potential values, indicating potential destabilization [51]. This is likely due to enhanced droplet fragmentation increasing attractive interactions, which dominate over repulsive forces when particles get close enough, leading to aggregation and emulsion instability [52].

However, in some formulations, zeta potential values remained similar or became less negative after the second cycle. This may be linked to surfactant concentration and overall system composition. Tween 80 plays a crucial role in stabilizing nanoemulsions, and its capacity to cover the surface area of droplets generated during additional processing may be limited [53]. Variability in zeta potential after the second cycle may indicate that the surfactant is no longer able to stabilize all the newly formed droplets, resulting in less negative zeta values and lower electrostatic stabilization [54]. In some cases, minimal change in zeta potential between cycles suggests the system had already reached surface charge equilibrium after the first cycle and further processing did not significantly alter electrostatic interactions [55].

To further explore the role of temperature, samples were also stored at 4 °C and analyzed at the same intervals, while ensuring minimal disturbance between measurements. On day 0, samples were conditioned for four hours, as their temperature after microfluidization was close to 45 °C. The behavior of nanoemulsions under these refrigeration conditions is shown in Table A2, indicating that increasing pressure led to smaller particle sizes in both temperature conditions, with most results ranging between 150 and 190 nm [56]. However, emulsions stored at 4 °C and processed at 5000 psi tended to show larger droplet sizes compared to those stored at room temperature, suggesting that low temperatures may affect size reduction efficiency or stabilization mechanisms [57].

In terms of zeta potential, refrigerated samples often showed more negative values, reaching up to −36 mV in some runs, which could suggest enhanced electrostatic repulsion under cold storage [58]. This behavior may be associated with reduced thermal motion at lower temperatures [59]. However, this trend was not consistent across all conditions, as some samples processed at lower pressures exhibited less negative zeta potentials, indicating a higher tendency toward aggregation.

Dispersity trends were generally consistent across both temperature conditions, with lower values at higher processing pressures, reflecting narrower droplet distributions [60]. Nevertheless, refrigerated emulsions tended to exhibit higher dispersity values than their room temperature counterparts, especially at lower pressures.

Importantly, despite some indications of improved electrostatic stabilization, visible creaming was observed in refrigerated samples, particularly at lower pressures. This suggests that refrigeration may negatively affect the overall physical stability of the system, highlighting that zeta potential alone is not sufficient to fully describe stability in these emulsions [61].

### 3.2. Optimization of Formulation and Improved Operating Conditions

Using Design Expert V10.0.7 software, experimental data collected from day 0 to day 60 under predefined storage and processing conditions were input for modeling. ANOVA models were analyzed for each response variable (droplet size distribution, zeta potential, and dispersity (D) to determine the significance of mean differences and identify the most influential input factor [62].

Table A3 highlights the *p*-values for the ANOVA models, color-coded in green to indicate statistical significance for specific experimental runs. Based on this analysis, experimental conditions were selected for further response surface modeling and optimization of input variables.

The analysis showed that after 60 days, no statistically significant differences were detected in the response variables, indicating the system had reached thermodynamic equilibrium [63]. By contrast, at day 30, significant differences were found, making this time point ideal for assessing the evolution of the response variables in relation to input parameters [64].

Among all variables, microfluidization pressure had the greatest influence on both droplet size and dispersity. Higher pressure induced greater shear forces, reducing droplet size and increasing interfacial area, which promoted system stability and homogeneity [65]. Dispersity values < 0.1 denote monodisperse systems optimal for pharmaceutical applications [66]. A Dispersity between 0.1 and 0.2 suggests moderate homogeneity, while values > 0.2 indicate higher variability [67]. The current formulations yielded dispersity’s ranging from 0.128 to 0.403.

Zeta potential analysis (Table A3) demonstrated that both pressure and avocado oil concentration significantly influenced surface charge. Increased pressure made the potential more negative, suggesting stronger electrostatic repulsion and hence better colloidal stability [68]. Changes in oil concentration affected droplet interface characteristics by altering Tween 80 orientation and surface charge [69]. A low *p*-value for oil concentration (*p* = 0.0176) suggested that excessive oil could reduce surface charge density and destabilize emulsions [70]. CBD concentration, on the other hand, had minimal impact on zeta potential, likely because CBD resides in the oil phase rather than at the interface [71].

Based on these findings, the following parameters were selected for in-depth modeling:

- Stability evaluation day: Day 30
- Storage temperature: 20 °C
- Microfluidization cycles: 1 cycle

Day 60 was excluded from ANOVA modeling due to a lack of significant variation, though it was retained for long-term stability interpretation. Refrigerated storage was also discarded due to observed creaming and added energy costs [72]. The selection of one cycle was justified by sufficient model fitting (R^2^ = 0.75) and reduced energy demand [73].

**Figure 4:**
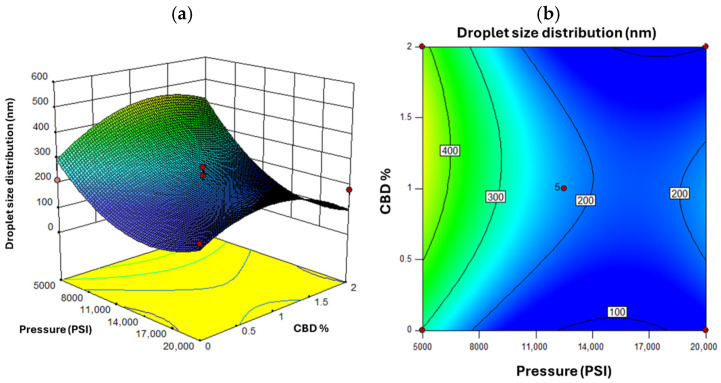
Response Surface for Droplet Size Distribution, where (**a**) represents the three-dimensional surface and (**b**) the two-dimensional surface with oil concentration as the fixed factor. Red dots in the figure represent the midpoints and extreme points of the Box-Behnken.

**Figure 5:**
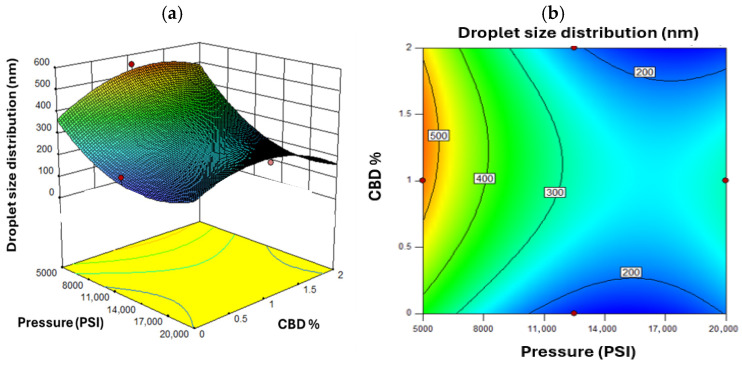
Response Surface for Droplet Size Distribution, where (**a**) represents the three-dimensional surface and (**b**) the two-dimensional surface with oil concentration at a value of 10% *w*/*w*. Red dots in the figure represent the midpoints and extreme points of the Box-Behnken.

Figure 4 shows the response surface for droplet size. As pressure decreased, droplet size increased especially at lower CBD concentrations. This aligns with the principle that higher pressure yields finer emulsions [74]. A non-linear response was observed for CBD concentration. Initial increases in CBD reduced droplet size, but beyond a threshold, viscosity effects limited further size reduction [75]. Figure 5 shows that higher oil concentrations under low pressure led to larger droplets, increasing the risk of instability [76]. The optimal pressure range was identified between 11,000 and 15,000 PSI, balancing energy cost with droplet size reduction. Beyond 15,000 PSI, marginal size improvements did not justify increased energy use [77].

**Figure 6:**
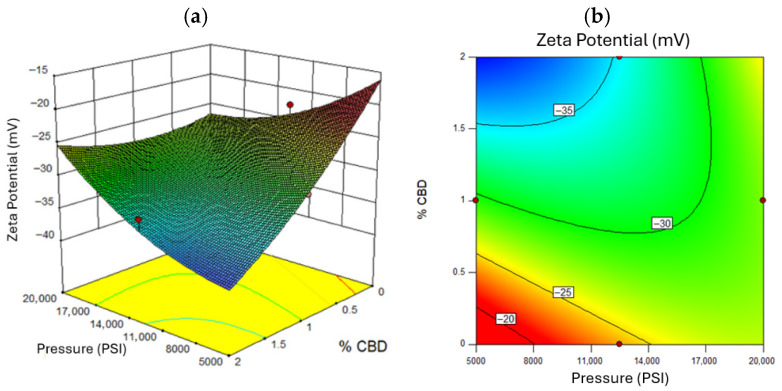
Response Surface for Zeta Potential, where (**a**) represents the three-dimensional surface and (**b**) the two-dimensional surface with oil concentration as the fixed factor. Red dots in the figure represent the midpoints and extreme points of the Box-Behnken.

**Figure 7:**
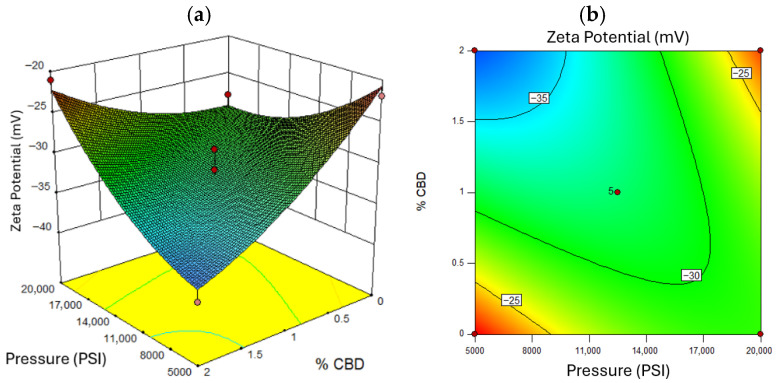
Response Surface for Zeta Potential, where (**a**) represents the three-dimensional surface and (**b**) the two-dimensional surface with oil concentration at a value of 10% *w*/*w*. Red dots in the figure represent the midpoints and extreme points of the Box-Behnken.

**Figure 8:**
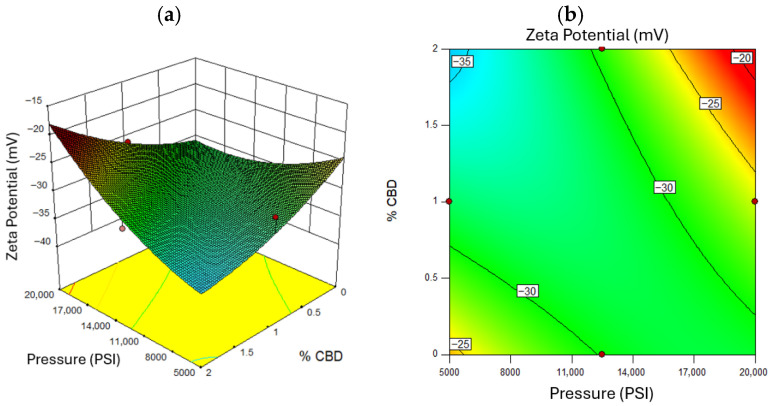
Response Surface for Zeta Potential, where (**a**) represents the three-dimensional surface and (**b**) the two-dimensional surface with oil concentration at a value of 8% *w*/*w*. Red dots in the figure represent the midpoints and extreme points of the Box-Behnken.

A zeta potential magnitude above 30 mV indicates good electrostatic stabilization [78]. Figure 6 confirms that higher pressures yielded more negative zeta potentials (~−35 mV), enhancing stability due to greater surface charge exposure [79]. Zeta potential also correlated with CBD concentration: higher levels increased the magnitude of repulsion forces [76]. Figure 6 shows that excessive oil concentration reduced surface charge, diminishing zeta potential [80]. Figure 7 and Figure 8 illustrates that optimal zeta potential at low oil concentrations occurred between 11,000 and 14,000 PSI and 1–2% CBD. Despite high zeta potential, droplet size remained too large for long-term stability [81].

Design Expert’s numerical optimization yielded a desirability value of 0.821, indicating a high degree of overall optimization. The optimal formulation was as follows:

- Pressure: 12,538 PSI;
- Avocado oil: 9.39% *w*/*w*;
- Tween 80: 3% *w*/*w*;
- CBD: 2% *w*/*w*;
- Water: balance to 100%.

This composition supports high stability and functionality [15]. Table 1 summarizes the fitted model coefficients for the three response variables, including *p*-values, degrees of freedom, and model performance metrics (R^2^, adjusted R^2^). Based on this analysis, the optimal nanoemulsion formulation was defined as the set of processing conditions that achieved the most favorable combination of physicochemical properties, particularly minimized droplet size, low dispersity, and adequate zeta potential, ensuring enhanced colloidal stability.

### 3.3. Encapsulation Efficiency Determination

The quantification of cannabidiol (CBD) in the nanoemulsion was performed using an external calibration curve constructed from standard solutions with concentrations ranging from 0.008 to 0.040 mg/mL [82]. The calibration curve exhibited excellent linearity, with a determination coefficient (R^2^) of 0.9922, thereby validating the reliability of the quantification method [83]. Based on this analysis, the encapsulation efficiency (EE) of the CBD nanoemulsion was determined to be 81.4% ± 1.5 indicating a highly effective entrapment of cannabidiol within the oil phase of the nanoemulsion system [84]. This high EE highlights the strong affinity of CBD for lipid environments, in line with its lipophilic nature and poor solubility in aqueous media [75]. The result suggests that a significant portion of the active compound was retained within the dispersed oil droplets rather than partitioning into the aqueous continuous phase [85].

This efficiency is especially relevant considering the physicochemical characteristics of CBD. As a hydrophobic molecule, CBD exhibits a high degree of solubility in medium-chain triglycerides and other non-polar or slightly polar solvents, which facilitates its retention within the internal phase of oil-in-water nanoemulsions [86]. Such behavior is supported by [87], who noted that cannabinoids tend to partition almost exclusively into the oil phase when properly emulsified [88].

The observed encapsulation efficiency lies within the typical range reported for cannabinoid nanoemulsions, generally between 70 and 90%, depending on formulation and process variables. As noted by [89], efficiencies in this range indicate that the formulation is robust, with proper selection of surfactants, oil phase, and processing conditions [90]. In the present case, the droplet size and Dispersity (D) closely mirrored those reported for optimized formulations, reinforcing the idea that the nanoemulsion matrix was stable and uniform, two critical parameters that support efficient encapsulation [91].

A key element influencing EE is the droplet size distribution, which directly affects the surface-to-volume ratio of the internal oil phase [92]. Smaller and more uniform droplets, indicated by a low dispersity, tend to provide better physical stability and reduce the diffusion of CBD out of the oil phase. The uniformity also ensures consistent CBD distribution throughout the system, minimizing potential concentration gradients that could lead to compound migration [93].

The relatively low standard deviation (±1.5%) further confirms the reproducibility and reliability of the encapsulation process, pointing to the consistency of the nanoemulsion system [94]. Additionally, the high encapsulation efficiency suggests limited degradation or loss of CBD during processing, an important consideration for thermally or oxidatively sensitive compounds [95].

This result also has significant implications for controlled release and bioavailability. A higher EE indicates that more of the active compound is retained within the delivery system, enabling sustained release over time [96]. Moreover, encapsulation within nano-sized oil droplets can protect CBD from degradation due to environmental factors such as light, oxygen, or enzymatic activity—an essential requirement for pharmaceutical or nutraceutical applications [97].

Furthermore, the strong performance of encapsulation results in an effective interaction between the oil phase and the surfactant system [98]. Tween 80, with its appropriate hydrophilic–lipophilic balance (HLB), likely formed a stable interfacial layer that both minimized coalescence and provided an efficient barrier to CBD diffusion. This interfacial stabilization plays a crucial role in preserving the encapsulated compound and maintaining the physical stability of the formulation [99].

The data also suggest that microfluidization parameters—although not discussed here—were adequately optimized to yield small and stable droplets that promote high EE [100]. Excessively large droplets or unstable emulsions tend to release the encapsulated compound more readily, leading to lower observed efficiencies. Thus, achieving this level of encapsulation indicates that the process conditions successfully preserved droplet integrity [101].

### 3.4. Thermal Behavior of Optimal Nanoemulsion

A differential scanning calorimetry (DSC) analysis was performed to compare the optimized nanoemulsion, a control nanoemulsion without CBD, and the wall material, which in this case corresponds to avocado oil.

**Figure A1:**
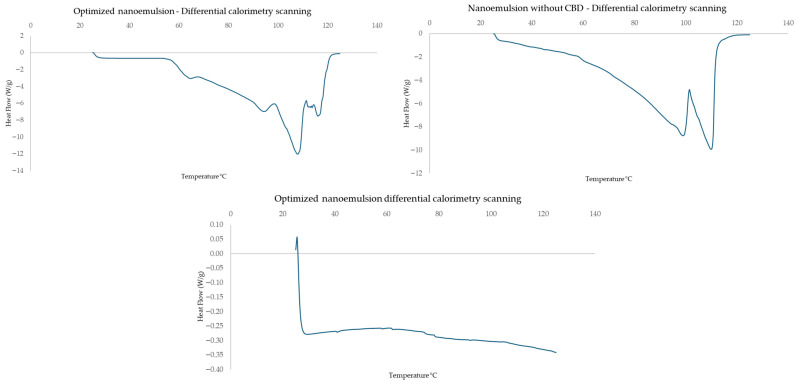
Differential Scanning Calorimetry (DSC) Thermograms and Thermal Transition Profiles of the Optimized CBD Nanoemulsion, Control Nanoemulsion, and Avocado Oil as Wall Material.

Initially, as shown in Figure A1, the wall material exhibits low sensitivity to temperature variations, which is consistent with the expected thermal profile of avocado oil based on its chemical composition. In contrast, the nanoemulsions display characteristic thermal peaks associated with their complex chemical composition.

Both nanoemulsion thermograms show a slight decrease in heat flow, which may be attributed to transitions related to the presence of Tween 80 or the aqueous phase. This behavior could indicate thermal relaxation phenomena or the loss of bound water within the system.

At approximately 60 °C, the first endothermic transition is observed. This peak may correspond to the melting of lipid components present in avocado oil or to specific interactions involving CBD, which influence the thermal behavior of the system. A comparison between the optimized nanoemulsion and the control reveals the absence of this endothermic peak in the latter, suggesting that this transition is associated with the presence of CBD.

In the temperature range between 70 and 90 °C, a continuous endothermic transition is observed, which may represent the continuation of the previous event or the melting of different phases within the nanoemulsion system.

Both nanoemulsions exhibit a minimum peak at approximately 94 °C, corresponding to an exothermic event. This behavior may be attributed to molecular-level restructuring processes within the nanoemulsion, potentially involving lipid components transitioning from an amorphous to a more crystalline arrangement.

Subsequently, an endothermic peak is observed at around 100 °C, which is likely associated with the evaporation of residual components such as water or solvents present in the formulation. However, the slight differences in peak intensity and shape in the CBD-containing nanoemulsion suggest that CBD may influence these thermal transitions through specific interactions with the lipid phase.

### 3.5. Release Kinetics

**Figure 9:**
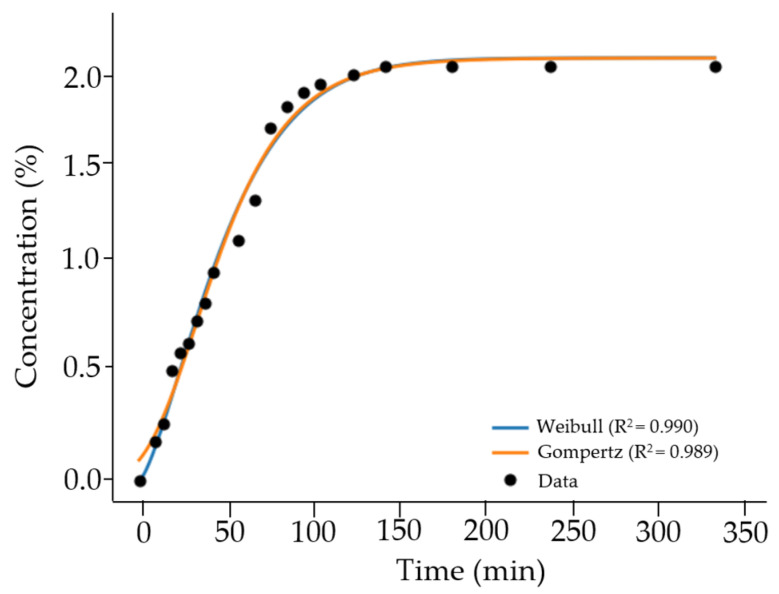
Graphical analysis of drug release kinetics fitted to different mathematical models. Experimental data are compared with model predictions.

The release profile of cannabidiol (CBD) from the optimized nanoemulsion exhibited a sigmoidal behavior, commonly associated with multi-stage release systems [102]. As shown in Figure 9, the release process consisted of an initial rapid phase followed by a gradual deceleration until reaching a plateau, with the final concentration approaching 2% of the initial CBD content, indicating depletion of the releasable fraction [103].

The release data were fitted to several kinetic models to better describe the system behavior. Among these, the Weibull and Gompertz models provided the best fit, both yielding high coefficients of determination (R^2^ = 0.990), highlighting their suitability for representing sigmoidal release profiles [104]. These models effectively capture systems in which the release rate is not constant but evolves over time due to changes in concentration gradients and system structure [105].

The good agreement with the Weibull model suggests a release behavior that cannot be described by a single mechanism, while the Gompertz model adequately represents the asymmetric sigmoidal trend observed experimentally [106,107]. In contrast, traditional models such as Higuchi (R^2^ = 0.841) and Korsmeyer–Peppas (R^2^ = 0.851) showed lower predictive capacity, indicating that simplified diffusion-based approaches are insufficient to describe the release behavior of nanoemulsion systems [108].

The relatively fast release observed compared to polymeric or gel-based systems may be attributed to the inherent characteristics of nanoemulsions, including reduced droplet size, increased interfacial area, and lower viscosity, which facilitate mass transfer [107,109].

### 3.6. Cell Viability

The cytotoxicity assay, designed to assess potential cell death, was conducted using the HaCaT keratinocyte cell line, a well-established model for evaluating skin-related formulations [85]. The primary objective was to determine whether the application of the optimized nanoemulsion, after 24 h of exposure, would exert any cytotoxic effects on the monolayer of HaCaT cells [91]. Additionally, a series of controls and alternative treatments were included to distinguish the effects of the formulation’s individual components, such as CBD, oil, surfactant, and solvents, on cellular behavior and viability [91].

**Figure 10:**
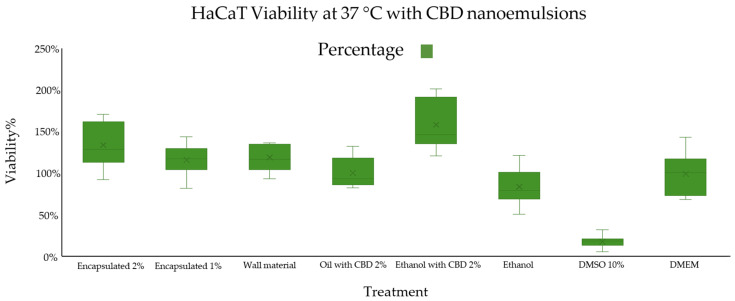
Cell viability of HaCaT cells at 37 °C after 24 h of incubation with CBD nanoemulsions and controls (n = 12). Data are expressed as percentage viability. The X in the box is the arithmetic mean.

As shown in Figure 10, eight treatment groups were evaluated under standardized conditions (37 °C, n = 12) to assess their effects on HaCaT cell viability. The results indicated that the treatment containing ethanol and 2% CBD yielded the highest apparent viability, reaching approximately 159% ± 29%, exceeding the control group [105]. While this outcome may suggest an increase in cellular activity, values above 100% should be interpreted with caution, as the MTT assay does not directly quantify cell proliferation but instead reflects mitochondrial metabolic activity [88]. Under certain experimental conditions, this assay may overestimate viability due to elevated enzymatic reduction in the tetrazolium salt, which is not necessarily associated with an increase in cell number [89]. Consequently, the observed increase is more appropriately attributed to enhanced metabolic activity rather than conclusive evidence of increased cell proliferation [106].

Free CBD dissolved in ethanol likely exhibits enhanced solubility and bioavailability, allowing for rapid cellular uptake through the lipid bilayer [110]. Ethanol, as a polar organic solvent, facilitates the transport of hydrophobic molecules like CBD across cell membranes [111]. Once internalized, CBD may participate in several intracellular signaling pathways associated with antioxidant response, mitochondrial activity, and energy metabolism, thereby acting as a bioenergetic enhancer [112]. These pathways include the modulation of endocannabinoid receptors (CB1 and CB2), MAPK/ERK signaling, and potential interaction with PPARγ, which have all been implicated in cell proliferation and survival [113].

Moreover, ethanol itself may act as a permeation enhancer, disrupting membrane lipid packing and facilitating the transport of solutes [114]. In the presence of CBD, this effect is magnified, leading to high intracellular concentrations of CBD and possibly explaining the unusually high viability percentages observed—well above 100% [115].

In contrast, the treatment with 10% DMSO exhibited the lowest cell viability, around 18% ± 6%, highlighting its cytotoxic nature [116]. DMSO, while commonly used as a solvent and cryoprotectant in low concentrations (<0.5%), is known to be toxic at higher concentrations [117]. It can disrupt cellular membranes, denature proteins, interfere with calcium homeostasis, and impair mitochondrial function, ultimately triggering apoptotic or necrotic pathways [118]. The sharp decline in viability observed in this group validates DMSO’s role as a positive cytotoxic control, demonstrating the sensitivity and reliability of the HaCaT model for detecting toxicity [119].

The treatments with optimal nanoemulsion containing 1% CBD and 2% CBD displayed notable but moderate increases in cell viability, recorded as 117% ± 17% and 135% ± 25%, respectively. These results confirm that nanoemulsion formulation does not induce cytotoxic effects and, instead, supports cell survival and moderate proliferation [120]. Importantly, they suggest that although the encapsulation of CBD slows its release, some CBD molecules remain accessible to the cells, likely due to incomplete encapsulation or surface-adsorbed CBD [121]. This controlled availability simulates a sustained-release mechanism, which may be beneficial in therapeutic applications requiring prolonged action and reduced systemic exposure [122].

The nanoemulsion without CBD served as a blank formulation control. Its viability level, which did not differ significantly from the untreated control, further confirms that the carrier system itself (oil, surfactant, and water) is biocompatible and non-toxic, meeting a critical requirement for any drug delivery vehicle [123]. The treatment with CBD in oil without encapsulation yielded intermediate results, reinforcing the idea that free CBD is beneficial for cell viability, but its efficacy depends on solubility and dispersion [98]. In oil, CBD’s hydrophobic nature limits its dispersion in the aqueous cell culture medium, reducing its cellular availability when compared to an ethanol formulation [124].

The difference in efficacy between the nanoemulsion and ethanol formulation underscores the importance of releasing kinetics and formulation matrix in determining biological outcomes [125]. While ethanol enables immediate and complete bioavailability, nanoemulsion provides a gradual release, which, although less intense initially, ensures a lower cytotoxic risk and prolonged therapeutic window [126].

This phenomenon illustrates the trade-off between burst efficacy and sustained safety: ethanol with CBD delivers a strong but short-lived stimulus, while nanoemulsions ensure consistent exposure, potentially reducing the likelihood of cellular stress responses over time [127]. From a pharmaceutical standpoint, this aligns with formulation strategies where encapsulation is used to buffer active compound release, minimizing peak concentrations that could lead to side effects [128].

To determine whether the observed differences between treatments were statistically significant, an analysis of variance (ANOVA) followed by a Tukey post hoc test was conducted [129]. As can be observed in Table 2, the 10% DMSO treatment showed highly significant differences compared to all other groups (*p*-value = 0.0000028), corroborating its cytotoxic profile [95]. The ethanol with 2% CBD treatment also exhibited statistically significant differences (*p*-value = 0.00038) relative to the remaining groups, due to its pronounced proliferative effect [130].

Notably, the treatments involving optimal nanoemulsions, the CBD-in-oil formulation, the nanoemulsion without CBD, and the ethanol control did not exhibit statistically significant differences in cell viability [131]. This outcome indicates that none of these formulations exerted cytotoxic effects under the conditions tested, and all maintained cellular viability within physiologically acceptable limits [132]. These findings support the conclusion that both complete nanoemulsion and its individual constituents, when appropriately dosed, are non-toxic and demonstrate biocompatibility with HaCaT cells [133].

## 4. Conclusions

The stability of nanoemulsions is strongly influenced by the operating pressure applied during microfluidization. Higher pressures lead to more efficient droplet disruption, resulting in smaller and more uniform droplet sizes. This effect is evident in the observed decrease in droplet size and dispersity (D) with increased pressure and additional microfluidization cycles.

The stability study at room temperature (20 °C) and under refrigeration (4 °C) showed that storage temperature had a minimal effect on nanoemulsion stability. Both conditions resulted in similar droplet size, dispersity, and zeta potential. However, refrigeration caused creaming and instability over time, making it unsuitable. Although multiple microfluidization cycles further reduce droplet size and dispersity, the energy cost is high, and a single cycle is sufficient for a stable nanoemulsion. Response surface analysis identified 11,000 to 15,000 PSI as the optimal pressure range for small, uniform droplets, with higher pressures increasing energy consumption. Avocado oil and solid concentration also significantly affected droplet size and stability. Zeta potential, a key stability indicator, showed that a magnitude greater than 30 mV prevents coalescence. Oil and CBD concentrations were the most critical factors for zeta potential, and the encapsulation efficiency of CBD was approximately 81.4% ± 1.5%, indicating effective retention of CBD in the oil phase, making the nanoemulsion a promising delivery system.

Also, the cell viability assay demonstrated that the optimal nanoemulsion does not induce cell death and supports cell proliferation, particularly in the presence of CBD. Ethanol with 2% CBD resulted in the highest cell viability, while dimethyl sulfoxide (DMSO) significantly reduced cell viability. The nanoemulsion and its individual components did not show harmful effects on the cells, confirming its safety for further application.

## Figures and Tables

**Table 1:** Analysis of Variance (ANOVA) for Response Surface Models Evaluating the Effects of Process Variables on Droplet Size, Dispersity (D), and Zeta Potential.

	Particle Size (nm)			Dispersity			Zeta Potential (mV)		
	SS	df	*p*-Value	SS	df	*p*-Value	SS	df	*p*-Value
Model	2.39 × 10^−5^	7	0.0030	0.19	9	0.0168	304.61	9	0.0296
A	−120.81	1	0.0005	−0.047	1	0.0660	1.76	1	0.1120
B	22.26	1	0.3613	0.012	1	0.6024	−2.49	1	0.0370
C	16.42	1	0.4961	0.036	1	0.1422	0.20	1	0.8450
AB	−39.62	1	0.2569	−0.035	1	0.2995	6.01	1	0.0032
AC	-	-	-	0.129	1	0.0041	−0.97	1	0.5001
BC	-	-	-	−0.023	1	0.4748	−2.87	1	0.0745
A^2^	118.73	1	0.0048	0.095	1	0.016	2.61	1	0.0914
B^2^	−101.01	1	0.0114	−0.112	1	0.0073	1.93	1	0.1910
C^2^	52.60	1	0.1336	0.021	1	0.5165	0.51	1	0.7108
Lack of fit	34,109.30	5	0.0520	0.013	3	0.4198	32.98	3	0.2229
Pure error	4466.44	4		0.014	4		19.41	4	
R^2^	0.86			0.88			0.85		
Adj. R^2^	0.75			0.72			0.67		

**Table 2:** Fisher’s Multiple Comparison Test for Cytotoxicity Treatments: Statistical Differences Among Experimental Conditions.

Comparisons	*p*-Value	Comparisons	*p*-Value
B-A	0.7601	E-C	0.0005
C-A	0.9726	F-C	0.2521
D-A	0.0708	G-C	0.0000
E-A	0.0004	H-C	0.8769
F-A	0.0007	E-D	0.0000
G-A	0.0000	F-D	0.8212
H-A	0.0561	G-D	0.0000
C-B	1.0000	H-D	1.0000
D-B	0.8561	F-E	0.0000
E-B	0.0000	G-E	0.0000
F-B	0.0934	H-E	0.0000
G-B	0.0000	G-F	0.0000
H-B	0.8122	H-F	0.8639
D-C	0.9036	H-G	0.0000

## Data Availability

The data presented in this study are not publicly available due to confidentiality restrictions. The datasets contain sensitive information related to proprietary formulations and experimental conditions, and therefore cannot be disclosed. Access to the data may be granted by the corresponding author upon reasonable request and with permission of the relevant stakeholders.

## Supplementary Materials

The following supporting information can be downloaded at: https://www.mdpi.com/article/10.3390/nano16080459/s1.

## Appendix A

**Table A1:** Physicochemical Characterization of Nanoemulsions at Day 0 and 20 °C after One and Two Microfluidization Cycles.

1 Microfluidization Cycle																	
Nanoemulsion	1	2	3	4	5	6	7	8	9	10	11	12	13	14	15	16	17
Pressure (psi)	5000	12,500	12,500	20,000	12,500	5000	12,500	12,500	12,500	20,000	12,500	5000	12,500	12,500	20,000	20,000	5000
Particle Size (nm)	190.2	167.7	154.1	150.5	159.9	185.3	158.8	161.7	158.8	151.3	146.7	212.0	204.1	160.3	146.2	152.3	237.2
ζ (mV)	−29.0	−22.2	−21.9	−31.1	−23.8	−28.5	−20.0	−19.5	−15.1	−33.1	−21.1	−23.5	−23.5	−22.6	−25.0	−28.6	−33.5
DISPERSITY	0.190	0.214	0.190	0.132	0.143	0.243	0.163	0.177	0.162	0.183	0.135	0.325	0.275	0.164	0.185	0.161	0.268
2 microfluidization cycle																	
Nanoemulsion	1	2	3	4	5	6	7	8	9	10	11	12	13	14	15	16	17
Pressure (psi)	5000	12,500	12,500	20,000	12,500	5000	12,500	12,500	12,500	20,000	12,500	5000	12,500	12,500	20,000	20,000	5000
Particle Size (nm)	195.1	146.6	146.5	157.5	149.6	179.4	149.9	150.6	163.8	154.8	143.2	180.6	147.1	151.1	140.7	145.9	290.7
ζ (mV)	−33.9	−28.9	−26.8	−30.9	−13.9	−17.5	−20.8	−12.0	−13.7	−18.3	−16.4	−27.7	−20.3	−14.2	−24.2	−27.7	−31.9
Dispersity	0.217	0.125	0.150	0.200	0.134	0.183	0.123	0.131	0.202	0.225	0.128	0.191	0.133	0.144	0.186	0.137	0.403

**Table A2:** Physicochemical Characterization of Nanoemulsions on Day 0 at 20 °C after One and Two Microfluidization Cycles.

1 Microfluidization Cycle																	
Nanoemulsion	1	2	3	4	5	6	7	8	9	10	11	12	13	14	15	16	17
Pressure (psi)	5000	12,500	12,500	20,000	12,500	5000	12,500	12,500	12,500	20,000	12,500	5000	12,500	12,500	20,000	20,000	5000
Particle size (nm)	198.8	156.0	154.7	155.3	159.4	185.4	156.5	159.6	159.5	148.6	151.2	186.2	158.5	159.9	146.7	160.1	235.3
ζ (mV)	−36.1	−33.7	−27.1	−34.4	−25.8	−25.8	−16.2	−20.3	−21.1	−22.7	−25.8	−26.6	−16.6	−19.6	−32.9	−34.1	−31.3
Dispersity	0.249	0.161	0.169	0.178	0.173	0.222	0.169	0.145	0.147	0.156	0.158	0.208	0.184	0.150	0.155	0.153	0.269
Creaming (mm)	0.2	0	0.1	0.1	0	0.2	0	0	0.1	0	0.1	0.1	0	0	0	0.1	0.3

**Table A3:** ANOVA Analysis of Nanoemulsion Physicochemical Properties (ASD, Zeta Potential, and DISPERSITY) at 20 °C Under 1 and 2 Microfluidization Cycles Over Time.

Conditions			ASD (nm)				Zeta Potential (mV)				Dispersity			
Cycles	Temperature	Day	Model	Pressure PSI [A]	%CBD [B]	% Oil [C]	Model	Pressure PSI [A]	%CBD [B]	% Oil [C]	Model	Pressure PSI [A]	%CBD [B]	% Oil [C]
1	4	0	0.0021	0.0001	0.4200	0.0258	0.2572	0.7955	0.6835	0.4114	0.0013	0.0001	0.1360	0.0778
1	4	8	0.0064	0.0003	0.9534	0.1893	0.0080	0.1176	0.0578	0.0022	0.3502	0.0958	0.4458	0.7244
1	4	15	0.0020	0.0002	0.0876	0.0434	0.0403	0.6844	0.0152	0.0667	0.0621	0.1378	0.2221	0.2812
1	4	30	0.0111	0.0005	0.5115	0.8272	0.0006	0.0005	0.1307	0.5127	0.3808	0.1658	0.7381	0.3231
1	4	60	0.1250	0.7434	0.8819	0.0857	0.2984	0.2126	0.5094	0.1911	0.1784	0.5852	0.2650	0.1110
2	4	0	0.0226	0.0017	0.3995	0.1545	0.0298	0.5779	0.8769	0.6326	0.0988	0.0983	0.5675	0.3184
2	4	8	0.0404	0.0049	0.5421	0.0762	0.0517	0.1525	0.4309	0.0760	0.3903	0.2526	0.9957	0.2013
2	4	15	0.0001	0.0001	0.2313	0.0176	0.0399	0.1414	0.0221	0.1302	0.0049	0.0052	0.0836	0.0277
2	4	30	0.6941	0.2549	0.9484	0.8223	0.2647	0.7966	0.1698	0.0892	0.5535	0.1823	0.7353	0.7849
2	4	60	0.1588	0.4270	0.8783	0.1201	0.0941	0.0255	0.6143	0.2799	0.2035	0.7100	0.9751	0.2391
1	20	0	0.0041	0.0002	0.2644	0.0176	0.0229	0.6869	0.4041	0.3327	0.0723	0.2677	0.8124	0.7497
1	20	8	0.0522	0.0084	0.5637	0.3180	0.0125	0.1983	0.0309	0.0013	0.1818	0.0899	0.5330	0.3995
1	20	15	0.0206	0.0017	0.2534	0.4799	0.0723	0.9743	0.1298	0.1517	0.0001	0.0003	0.0004	0.0307
1	20	30	0.0173	0.0018	0.3977	0.5278	0.0633	0.2200	0.0939	0.8867	0.0333	0.0967	0.6472	0.1905
1	20	60	0.0042	0.0002	0.2656	0.6968	0.1558	0.1689	0.5012	0.1329	0.0547	0.0092	0.8761	0.4839
2	20	0	0.0186	0.0027	0.6035	0.0949	0.3853	0.6134	0.8345	0.3349	0.1757	0.5274	0.0753	0.2336
2	20	8	0.0112	0.0022	0.0120	0.5337	0.1427	0.5691	0.9047	0.0822	0.0060	0.0008	0.0535	0.5583
2	20	15	0.0055	0.0004	0.2052	0.0476	0.0407	0.5572	0.1680	0.0529	0.1290	0.0235	0.8714	0.1940
2	20	30	0.0559	0.0090	0.8092	0.5758	0.0295	0.8472	0.5811	0.0042	0.1637	0.0418	0.3814	0.7737
2	20	60	0.0052	0.0005	0.0271	0.5282	0.1563	0.4704	0.9252	0.5752	0.0246	0.0046	0.2873	0.1722
